# Therapeutic benefit of combining calorie-restricted ketogenic diet and glutamine targeting in late-stage experimental glioblastoma

**DOI:** 10.1038/s42003-019-0455-x

**Published:** 2019-05-29

**Authors:** Purna Mukherjee, Zachary M. Augur, Mingyi Li, Collin Hill, Bennett Greenwood, Marek A. Domin, Gramoz Kondakci, Niven R. Narain, Michael A. Kiebish, Roderick T. Bronson, Gabriel Arismendi-Morillo, Christos Chinopoulos, Thomas N. Seyfried

**Affiliations:** 1 Department of Biology, Boston College, Chestnut Hill, MA 02467 USA; 2 BERG LLC, Framingham, MA 01701 USA; 3 Mass Spectrometry Center, Chemistry Department, Boston College, Chestnut Hill, 02467 USA; 4 Rodent Pathology Core, Harvard Medical School, Boston, MA 02215 USA; 5 Facultad de Medicina, Instituto de Investigaciones Biológicas, Universidad del Zulia, 526 Maracaibo, Venezuela; 6 Department of Medical Biochemistry, Semmelweis University, Budapest, 1094 Hungary

**Keywords:** Cancer metabolism, CNS cancer

## Abstract

Glioblastoma (GBM) is an aggressive primary human brain tumour that has resisted effective therapy for decades. Although glucose and glutamine are the major fuels that drive GBM growth and invasion, few studies have targeted these fuels for therapeutic management. The glutamine antagonist, 6-diazo-5-oxo-L-norleucine (DON), was administered together with a calorically restricted ketogenic diet (KD-R) to treat late-stage orthotopic growth in two syngeneic GBM mouse models: VM-M3 and CT-2A. DON targets glutaminolysis, while the KD-R reduces glucose and, simultaneously, elevates neuroprotective and non-fermentable ketone bodies. The diet/drug therapeutic strategy killed tumour cells while reversing disease symptoms, and improving overall mouse survival. The therapeutic strategy also reduces edema, hemorrhage, and inflammation. Moreover, the KD-R diet facilitated DON delivery to the brain and allowed a lower dosage to achieve therapeutic effect. The findings support the importance of glucose and glutamine in driving GBM growth and provide a therapeutic strategy for non-toxic metabolic management.

## Abstract (web summary)

Purna Mukherjee et al demonstrate that administering a glutamine analogue together with a calorie-restricted ketogenic diet can reduce brain tumour growth and inflammation, while enhancing survival, in two syngeneic orthotopic mouse models.

## Introduction

Glioblastoma (GBM) has the highest mortality rate among primary brain tumours in adults and remains largely unmanageable with a life expectancy of about 6–24 months following diagnosis. Despite decades of research, little progress has been made in improving GBM survival^1^. A defining characteristic of GBM is the secondary structures of Scherer, which includes diffuse parenchymal growth and invasion over the subpial surface, along white matter tracks, and through the Virchow-Robin space^2^. The highly invasive nature of GBM makes most current therapies ineffective. Most patients die from intracranial pressure due to inflammation, edema, and distal tumour cell invasion. Hence, the proliferative and invasive nature of GBM is the major hindrance for effective therapeutic intervention.

GBM contains a range of morphologically diverse neoplastic cell types that express glial, stem cell, and myeloid/mesenchymal markers^3–5^. Also recognized are abnormalities in the number, structure, and function of mitochondria in GBM tumour tissue^6–10^. In addition, recent studies show abnormalities in GBM mitochondrial-associated membranes, which are also critical for normal mitochondrial function^11^. Moreover, the content and composition of cardiolipin, the signature lipid of the mitochondrial inner membrane that regulates oxidative phosphorylation (OxPhos), shows deviations from the normal in five different murine GBM models^12^. As mitochondrial function and efficiency is dependent on structure^13^, OxPhos is expected to be lower in GBM than in normal brain tissue. Indeed, significant evidence shows that OxPhos is defective in human and murine GBM^7,12,14^.

Regardless of GBM cell type, glucose and glutamine are required for growth and invasion through glycolysis and glutaminolysis, respectively^15–19^. Although glucose is the primary metabolic fuel for neurons and glia, normal brain cells can transition to ketone bodies (primarily β-hydroxybutyrate) for energy under hypoglycemic conditions^20^. In contrast, GBM cells lack metabolic flexibility and are susceptible to death in response to reduced glucose bioavailability^21^. Although brain glutamine levels are tightly regulated via the glutamine-glutamate cycle, the bioavailability of glutamine increases following surgery, radiation, and chemotherapy, which fuels tumour growth^20,22^. If glucose and/or glutamine remain available, the tumour cells will utilize the fuel and grow, making long-term management difficult.

The calorically restricted ketogenic diet (KD-R) is a low carbohydrate, high fat diet, that reduces blood glucose and increases blood ketone bodies to therapeutic levels, while simultaneously inducing anti-inflammatory effects^23^. The primary ketone bodies, β-hydroxybutyrate and acetoacetate, are also neuroprotective and non-fermentable^24,25^. We found previously that the KD-R is a non-toxic anti-inflammatory approach to reduce pathology in experimental human and mouse brain tumours^23^. The neoplastic GBM cells can also synthesize high-energy phosphates through mitochondrial substrate level phosphorylation supported by glutaminolysis^25,26^. In addition, glutamine provides a continuous source of nitrogen for de novo synthesis of nucleotides and proteins in cancer cells^27^. The extent of glucose and glutamine metabolism in tumour cells depends not only on the tumour cell type, but also on the location and microenvironment.

Recently, the efficacy of simultaneous inhibition of both glycolysis and glutaminolysis has been demonstrated in experimental cancer models and in a human GBM case report^15,28,29^. Therapeutic success depends on modifications in dosage, scheduling, and timing in order to enhance efficacy while reducing toxicity^6^. 6-diazo-5-oxo-L-norleucine (DON) is a glutamine antagonist that was initially isolated from a Streptomyces broth in 1956. DON blocks multiple glutaminases, thus restricting key metabolites needed for glutaminolysis and the synthesis of nucleotides and proteins^30^. The anti-tumour efficacy of DON was confirmed in different tumour models, and in humans with various cancers^28–30^.

In the present study, we show a unique therapeutic response from the simultaneous targeting of glucose and glutamine using a KD-R and DON, respectively, in syngeneic orthotopic mouse models of GBM. The VM-M3 GBM tumour arose in the cerebral cortex of a mouse from the VM/Dk inbred strain, which has a high incidence of spontaneous brain tumours relative to other strains. The mesenchymal VM-M3 tumour cells invade distally throughout the brain using the secondary structures of Scherer^2^. In addition to the VM-M3 tumour, we also evaluated the effects of the KD-R and DON combination on the growth of the CT-2A brain tumour. The highly angiogenic CT-2A tumour was produced from 20-methancolanthrene in the cerebral cortex of a C57BL/6J mouse, and has several characteristics in common with the neoplastic neural stem cells found in human GBM^31,32^. Abnormalities in the electron transport chain were found in both tumours^12^. Our data show that the combination of KD-R and DON administration cause massive tumour cell death in both of these preclinical GBM mouse models and highlights the importance of targeting glycolysis and glutaminolysis simultaneously for the metabolic management of GBM.

## Results

The aim of this research was to determine if the simultaneous targeting of glucose and glutamine, while under therapeutic ketosis, could manage late-stage growth and enhance survival without toxicity in two different syngeneic mouse models of GBM. We administered DON 7 days after tumour implantation, which is considered late for the VM-M3/Fluc tumour, as neurological signs and symptoms first appear at 10 to 12 days post implantation with morbidity occurring in 100% of the tumour-bearing mice by 14–18 days^2^. Additional results were obtained in C57BL/6J inbred mice bearing the syngeneic CT-2A high-grade stem cell glioma^32^. DON, at the concentrations used in this study, had no toxic effects in non-tumour-bearing VM/Dk mice, as was shown previously for C57BL/6J mice^33^.

**Fig. 1:**
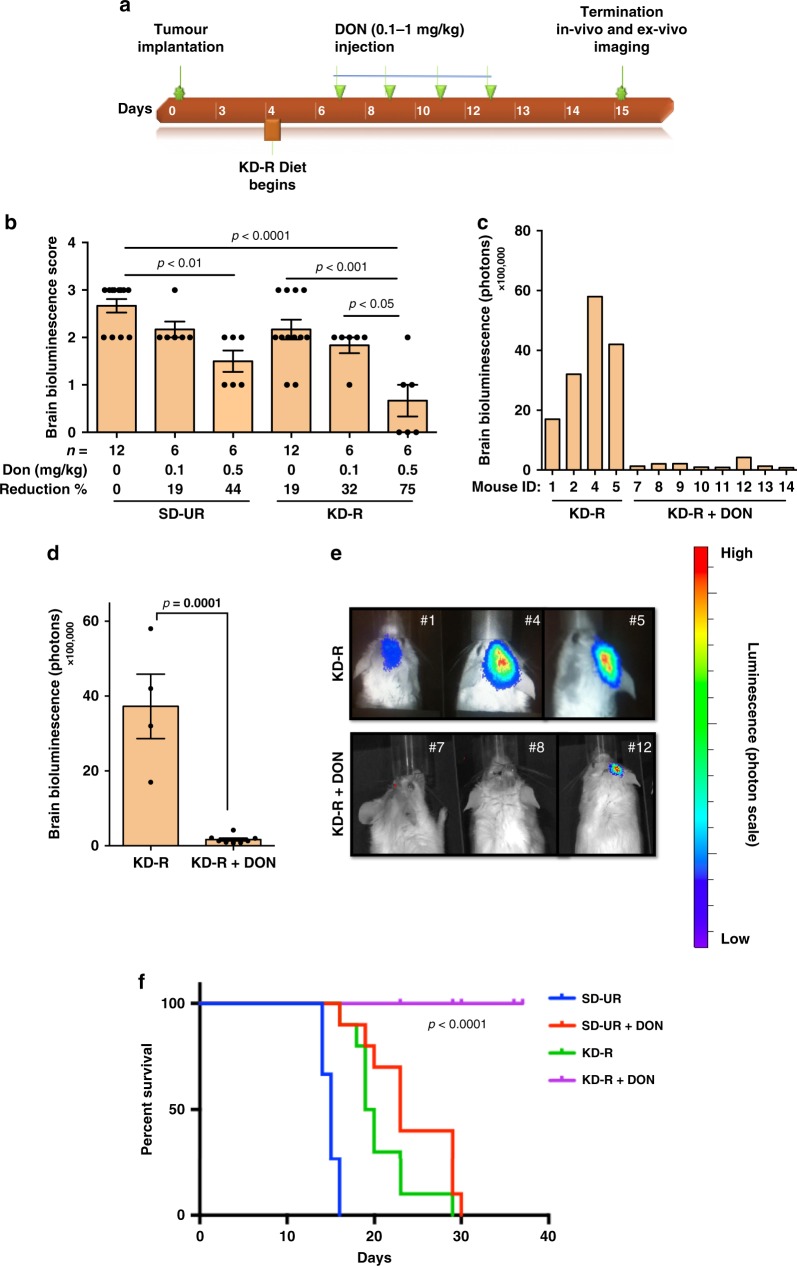
Restricted ketogenic diet with DON reduces progression and mortality of the VM-M3 GBM. VM/Dk inbred mice were implanted orthotopically with a small (1.0 mm × 1.0 mm) tissue fragment from the VM-M3 tumour on day 0. The implanted mice were divided into two groups on day 4 and were fed either a standard chow diet unrestricted or ad libitum (SD-UR), or a ketogenic diet (KD-R) in restricted amounts to reduce body weight by about 15%. DON (0.1–1.0 mg/kg) was injected i.p. 7 days following orthotopic tumour implantation. The diet feeding was continued and DON was injected every day or every alternate day as shown in (**a**). All mice were imaged in vivo and terminated on day 14 or 15 when all control mice appeared moribund (experiments 1, 2). For experiment 1, bioluminescence was scored from 0–4, following the administration of lower doses of DON (0.1 and 0.5 mg/kg). Values are expressed as the mean ± SEM and a one-way analysis of variance followed by Tukey’s post hoc test was performed to determine the significance between groups (**b**). In experiment 2, in vivo bioluminescent photon values were obtained following the administration of DON (1.0 mg/kg) in mice under the KD-R (**c**). The average in vivo bioluminescent photon values were calculated for the KD-R (*n* = 4 mice) and KD-R + DON (*n* = 8 mice) in experiment 2. Values are presented as the mean ± SEM and the *P* value was calculated using a two-tailed student’s *t*-test (**d**). In vivo bioluminescent images of three representative mice from both study groups in experiment 2 are shown in (**e**). For experiment 3, a survival study was performed and a Kaplan–Meier survival plot was configured for SD-UR (*n* = 15 mice), SD-UR + DON (*n* = 10 mice), KD-R (*n* = 10 mice), and KD-R + DON (*n* = 10 mice) (**f**). The log-rank statistical analysis test showed a significant difference between groups in this survival study. Source data are provided as  Supplementary Data 1

To observe the effect of the KD-R and DON on the VM-M3 tumour model, we conducted three consecutive experiments. Experiment 1 included four groups (SD-UR, SD-UR + DON, KDR, KDR + DON) with lower DON dosages (0.1 and 0.5 mg/kg). Experiment 2 involved both in vivo and ex vivo imaging of two study groups (KD-R and KD-R + DON) following the administration of a higher dose of DON (1.0 mg/kg). Experiment 3, a survival study, also used the higher DON dose (1.0 mg/kg) and included the four groups from Experiment 1 (SD-UR, SD-UR + DON, KDR, KDR + DON) (Fig. 1).

### KD-R + DON reduces VM-M3 burden and increases survival

The KD-R + DON treatment was performed as outlined in the study timeline (Fig. 1a). In experiment 1, two doses of DON (0.1 and 0.5 mg/kg) were used in both the SD-UR and KD-R groups (Fig. 1b). d-luciferin was injected intraperitoneally (i.p.) 15 days after tumour implantation, and in vivo bioluminescence imaging was performed. The total VM-M3 tumour bioluminescence photon values were scored from 0 to 4, with 0 being the baseline value and 4 being the highest value. Several mice from the SD-UR group that did not receive DON had a maximum photon score of 4, whereas the mice from the KD-R group + DON had a minimum photon score of 0 or 1. Compared to the control SD-UR group, mice receiving the KD-R alone or SD-UR + DON had a 19 and 44% lower photon value, respectively (Fig. 1b). The bioluminescence photon score was 75% lower in mice that received the KD-R + DON than in mice receiving each therapy alone, indicating a synergistic effect of the diet/drug combination.

Experiment 2 involved a larger number of mice (n = 8 mice) that were treated with a higher dose of DON (1.0 mg/kg). Due to the observed synergy between the KD-R and DON in experiment 1, only the KD-R group was selected for further experiments. All mice in this experiment expressed tumour burden by day 7. Individual brain bioluminescent values are shown in Fig. 1c. A baseline value of bioluminescence was seen in 7 out of the 8 KD-R fed mice that received DON and a reduction in average photon value was observed in the DON-treated mice (Fig. 1d). A representative number of mice from both study groups are shown in Fig. 1e. All untreated SD-UR control mice died between day 15 and 18. In contrast, mice treated with the diet/drug combination survived until day 40 and onwards (Fig. 1f).

A standard diet restricted (SD-R) group or a ketogenic diet unrestricted (KD-UR) group were not included in this study. We previously showed that a KD-R is more effective at reaching the target level of blood glucose and ketones than is a SD-R^23,34,35^. In other words, the ketone levels are higher and the glucose levels are lower under KD-R feeding than under SD-R feeding despite similar caloric restriction and reduced body weight. A KD-UR was also not included in this study, as consumption of ketogenic diets in unrestricted amounts can cause insulin insensitivity and weight gain, as we previously described^23,35,36^.

### KD-R + DON reduces ex vivo bioluminescence in VM-M3 brain

**Fig. 2:**
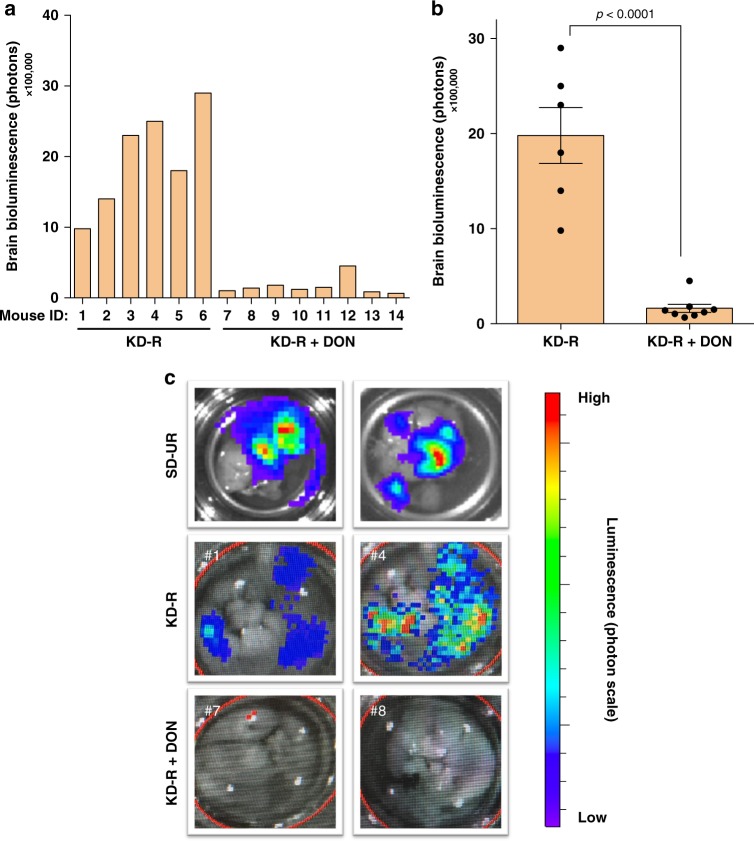
Restricted ketogenic diet with DON reduces VM-M3 GBM bioluminescence. VM/Dk mice were implanted with a VM-M3 tumour, as described in Fig. 1. Mouse brains were imaged ex vivo on day 14 or 15. Mice were imaged ex vivo after in vivo bioluminescent imaging, as described in Fig. 1. Individual ex vivo bioluminescent photon values were measured following the administration of DON (1.0 mg/kg) in KD-R mice (**a**). From the individual measurements, the average ex vivo bioluminescent photon value was calculated for both the KD-R (*n* = 6 mice) and KD-R + DON (*n* = 8 mice) groups. Values are presented as the mean ± SEM and significance of differences was determined following a two-tailed student’s *t*-test (**b**). Ex vivo bioluminescent images of brains from representative mice in experiment 2 are shown in comparison to SD-UR (**c**). Source data are provided as Supplementary Data 1

The VM mice from experiment 2 were sacrificed after in vivo imaging, and their brains were immediately resected and individually imaged ex vivo for total bioluminescence as described in the Methods. Ex vivo imaging provides a more accurate assessment of tumour progression than in vivo brain imaging due to the direct contact of the d-luciferin with the brain tissue and the removal of the blood–brain barrier as a limiting factor. The individual ex vivo bioluminescent values for the DON-treated mice were below the baseline value indicating a reduction in tumour cell viability (Fig. 2a). These values were consistent with the in vivo photon values (Fig. 1c). A reduction in average ex vivo photon value was also observed in the mice treated with DON alone (Fig. 2b). A representative number of brains from the KD-R and the DON-treated mice are shown in comparison to the SD-UR mice (Fig. 2c). It should be noted that an accurate in vivo bioluminescent value was not obtained for mouse #3 and #6 due to several variables during the imaging process (tumour burden, isoflurane exposure, blood collection, etc.). If an individual mouse did not provide an accurate in vivo bioluminescent reading, the brain was immediately resected, placed in sterile PBS and imaged ex vivo. For this reason, there are slight discrepancies in the number of mice imaged for in vivo bioluminescence and for ex vivo bioluminescence in experiment 2.

### KD-R + DON reduces proliferation and kills VM-M3 cells

**Fig. 3:**
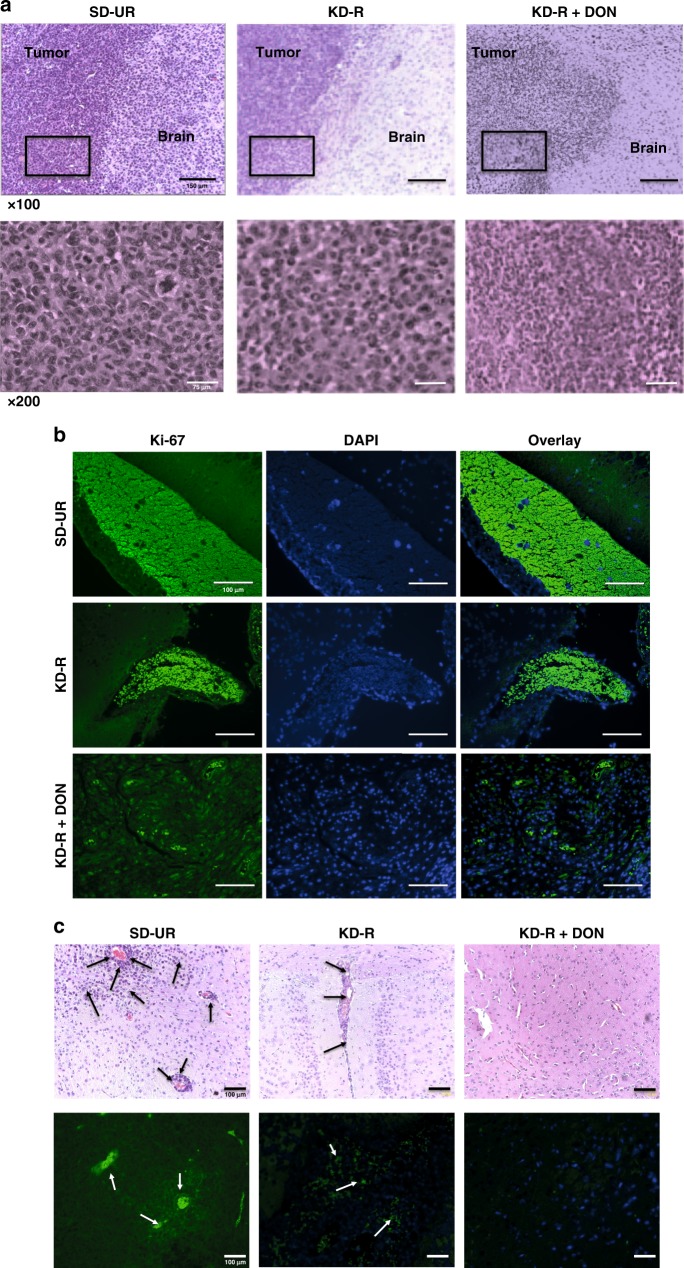
Restricted ketogenic diet with DON kills VM-M3 GBM cells in brain. VM/Dk mice were implanted with VM-M3 cells orthotopically in the brain on day 0 as described in Fig. 1. The brains were fixed in formalin for histology, processed, and stained as described in Methods. Histological analysis (H&E) was used to validate the presence of tumour cells (**a**). The top panel images show the core of the tumour mass growing in the brain. The black boxes in the top panel’s images are shown in higher magnification in the bottom panels. All scale bars are 150 μm for x100 and 75 μm for x200. Ki-67 positive nuclear staining (×100), as expressed by green fluorescent labeled cells are shown in (**b**). DAPI is used for the Ki-67 negative nuclear stain. The invasion of tumour cells in the brain tissue is evident (arrows) in both histological (H&E) analysis (×100) and Ki-67 staining (**c**). All scale bars for **b** and **c** are 100 μm

Histological evaluation of brain tumour tissue from mice with the VM-M3 tumour is shown in Fig. 3. Compared with the diffuse, ill-defined border of the VM-M3 tumour cells growing in the SD-UR brains, the tumour in the KD-R brains appeared less dense, less invasive, and had a more defined border (Fig. 3a). DON-treated brain tumours showed an area of dead cells in the primary tumour. In addition, the percentage of Ki-67 proliferating cells (green) was lower in the KD-R tumour than in the SD-UR tumour and was further reduced in the KD-R + DON-treated tumour tissue (Fig. 3b).

VM-M3 cells are highly invasive through the secondary structures of Scherer^2^. VM-M3 cells were observed invading from the core tumour into the normal appearing brain (Supplementary Fig. 1). Moreover, the extensive perivascular necrosis and subarachnoid invasion, hallmarks of human GBM (Supplementary Fig. 1), were clearly present in the VM-M3 model of GBM. The KD-R reduced the invasion of VM-M3 cells as shown in both H&E and Ki-67 staining (Figs. 3b, c). VM-M3 GBM tumour cells were seen invading the perivascular area in the SD-UR brain, whereas fewer invading GBM tumour cells were seen in the KD-R brains. No invading VM-M3 cells were seen in the KD-R + DON-treated brains (Fig. 3c). These findings indicate that the diet/drug therapy both kills tumour cells and inhibits invasion.

### KD-R facilitates DON delivery to the VM-M3 tumour

**Fig. 4:**
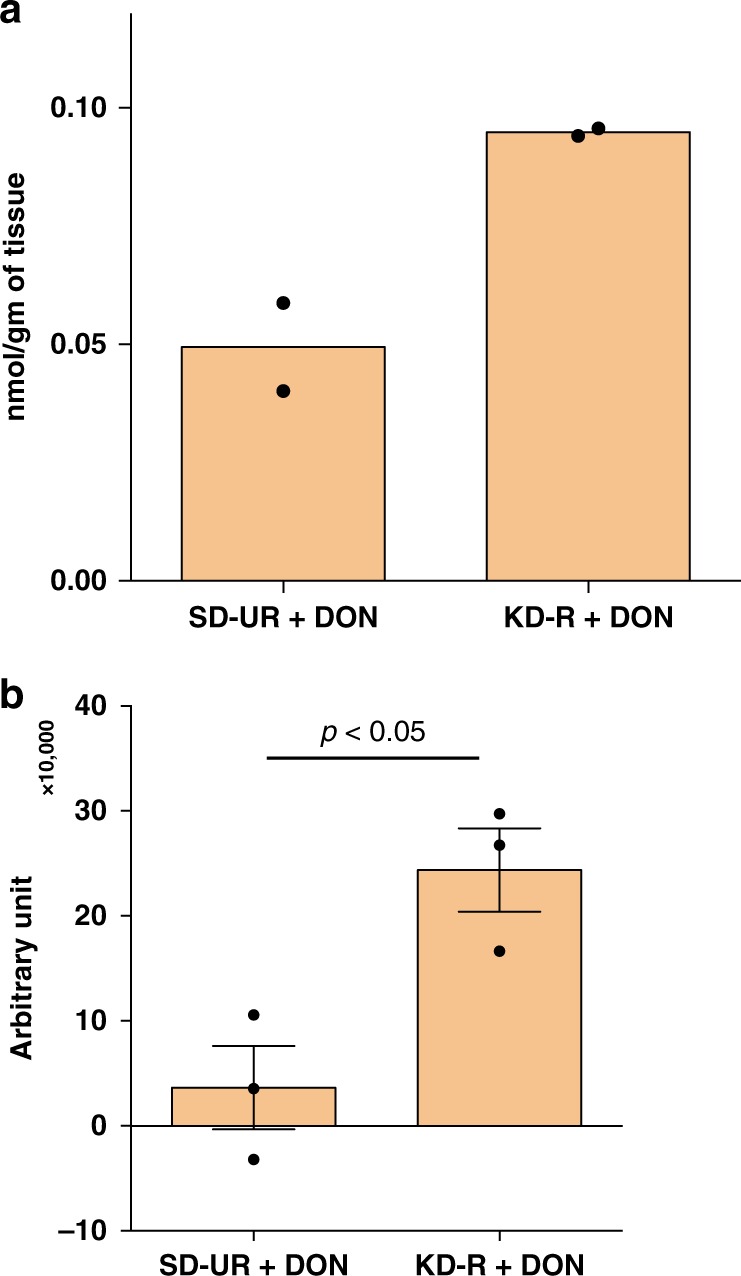
Restricted ketogenic diet increases DON delivery to the VM-M3 GBM. The content of DON in the VM-M3 brain tumour tissue was quantified using two LC/MS/MS procedures, as described in Methods. The brain tissue was analyzed for DON content 60 min after i.p. DON injection. The values in experiment **a** are presented as mean of two independent samples, while the values in experiment **b** are presented as the mean ± SEM (*n* = 3 mice). Following a two tailed student's t-test, the difference between the two groups in experiment **b** was significant (*P* *<* 0.05). Both analytical procedures showed that DON content in tumour tissue was greater under KD-R feeding than under SD-UR feeding. Source data are provided as Supplementary Data 1

Plasma and brain tissues were prepared for DON analysis one hour after systemic DON injection, as described in Methods. LC/MS/MS analysis showed that the concentration of DON in the VM-M3 tumour tissue was about two-fold greater in mice fed the KD-R than in mice fed the SD-UR (Fig. 4a). In another independent experiment, using a similar analytical system, the concentration of DON in the VM-M3 tumour tissue was about 3-fold greater in mice fed the KD-R than in mice fed the SD-UR (Fig. 4b). In both methods of measurement, DON delivery to the brain was greater in the mice fed the KD-R than in the mice fed the SD-UR (Fig. 4a, b). DON was detected in brain tissue 60 min following i.p. injection. These findings indicate that the KD-R facilitated delivery of DON to the VM-M3 tumour tissue.

### KD-R + DON reduces TNF-α expression and GKI

**Fig. 5:**
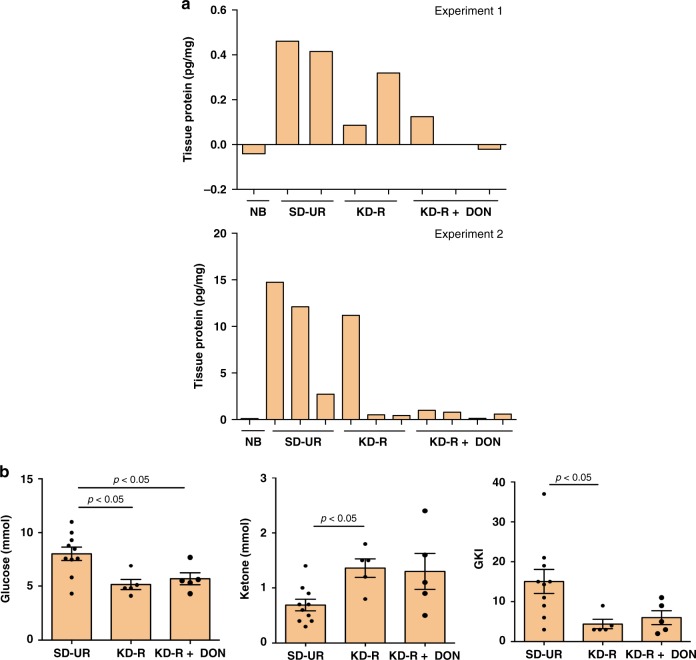
Restricted ketogenic diet with DON reduces TNF-α in the VM-M3 brain tumour tissue, and the Glucose Ketone Index (GKI) in the blood. ELISA was used to measure TNF-α in brain tumour tissue lysates in two different experiments, and the content was expressed as pg/mg of protein. Normal brain (NB) was used as a negative control tissue. The range of TNF-α concentration was −0.04–0.1 pg/mg for NB (*n* = 2 normal mouse brain tissue); 0.4–14.7 pg/mg for SD-UR (*n* = 5 mouse tumour brain tissue); 0.09–11.2 pg/mg for KD-R (*n* = 5 mouse tumour brain tissue); and 0.02–1.0 pg/mg for KD-R + DON (*n* = 7 mouse tumour brain tissue) (**a**). Data showing that blood glucose is lower and blood ketones are higher in mice fed the KD-R or KD-R + DON (*n* = 5 independent mouse blood samples per group) than in mice fed the SD-UR (*n* = 10 independent mouse blood samples). This shift in blood glucose and ketones causes a significant reduction in the GKI (**b**). Values are expressed as the mean ± SEM and a one-way analysis of variance followed by Tukey's post hoc test was performed to determine the significance between groups. Source data are provided as Supplementary Data 1

TNF-α expression was measured in lysates prepared from the right brain cortex that contained the VM-M3 tumour. These mouse brain samples are different from the mice used for imaging in Figs. 1 and 2. A high level of TNF-α expression, a biomarker for inflammation, was found in the right brain cortex of mice fed the SD-UR. Two independent analyses showed a similar trend in the TNF-α level (pg/mg of protein in the lysate). TNF-α expression was either low or undetectable in the normal brain tissue (Fig. 5a). TNF-α expression was lower in the brain of KD-R-fed mice than in the brain of SD-UR-fed mice, whereas TNF-α expression was even lower in the KD-R + DON-treated mice, suggesting a minimum amount of tumour load and reduced inflammation compared to the other two groups (Fig. 5a).

Reduced glucose and increased ketone body levels are blood biomarkers for the KD-R and evidence of therapeutic ketosis. The ratio of glucose to ketone bodies (Glucose Ketone Index, GKI) has been linked to the therapeutic efficacy of the ketogenic diet (KD) for brain cancer^36^. The lower the GKI value, the greater the metabolic stress on the tumour cells. The GKI values for the KD-R mice, treated and untreated with DON, were lower than for SD-UR mice. The GKI values in the KD-R + DON mice were similar to that in the KD-R mice, indicating that a low GKI was maintained under DON treatment (Fig. 5b).

### KD-R + DON reduces Iba-1 expression in VM-M3

**Fig. 6:**
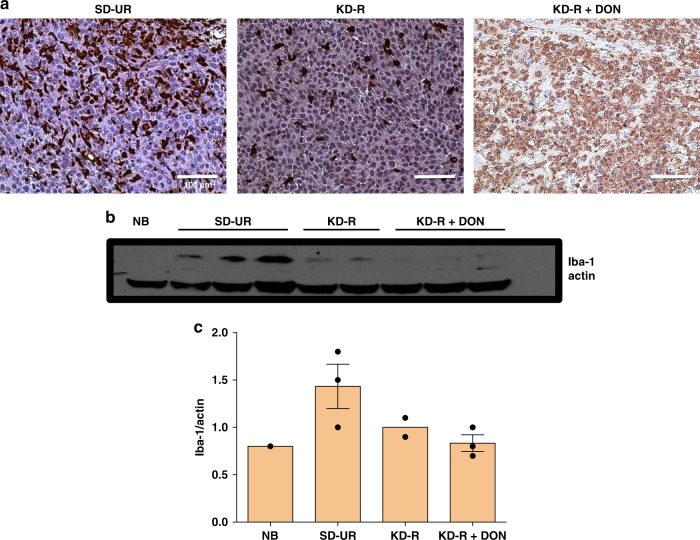
Restricted ketogenic diet with DON reduces the Iba-1 expression in the VM-M3 brain tumour tissue. Immunohistochemistry (IHC) of Iba-1 was performed in formalin fixed brain tissues as described in Methods. In comparison to all other groups, the expression of Iba-1 is highest in the SD-UR tumour as seen by the positively stained brown cells. Iba-1 IHC staining was noticeably less in the VM-M3 tumor than in the KD-R-fed mice and the DON-treated mice (**a**). All scale bars are 100 μm. Western blot analysis of Iba-1 protein expression in the tumours indicates a decrease in the expression of Iba-1 in the DON-treated tumours (*n* = 3 mouse tumour brain tissue) in comparison to SD-UR (*n* = 3 mouse tumour brain tissue). A decrease in expression was also seen for KD-R mice (*n* = 2 mouse tumour brain tissue). Normal brain (NB) was used as a negative control tissue (**b** and **c**). Source data are provided as Supplementary Data 1

Iba-1 protein expression was analyzed in the VM-M3 brain tumour tissue by immunohistochemistry and western blot. Iba-1 was heavily expressed in VM-M3 tumour and normal microglia/macrophages, as we described previously^37^. The KD-R reduced the expression of Iba-1 as shown in both immunohistochemistry and western blot (Fig. 6a, b). There was a reduction in Iba-1 expression in DON-treated brain tumour tissue. It is important to note that the majority of tumour cells in the DON-treated tumours were dead and therefore did not stain positively for nuclear methyl green (Fig. 6a). This finding is consistent with the histological observation found from our H&E analysis (Fig. 3). The reduced Iba-1 staining is also consistent with the reduced TNF-α expression (Fig. 5), suggesting reduced inflammation in the brains of both KD-R and DON-treated mice. The reduced Iba-1 histological staining was also consistent with the findings from western blot analysis of Iba-1 expression (Fig. 6b, c). Expression of Iba-1 in the brain tissue of the KD-R + DON-treated mice was lower than the expression in the untreated SD-UR mice, and was similar to the expression in normal non-tumour mouse brain. Iba-1 expression was also lower in the KD-R mice than in the SD-UR mice, but the reduction was not significant (Fig. 6b, c and Supplementary Fig. 2).

### KD-R + DON reduces edema in CT-2A and increases survival

**Fig. 7:**
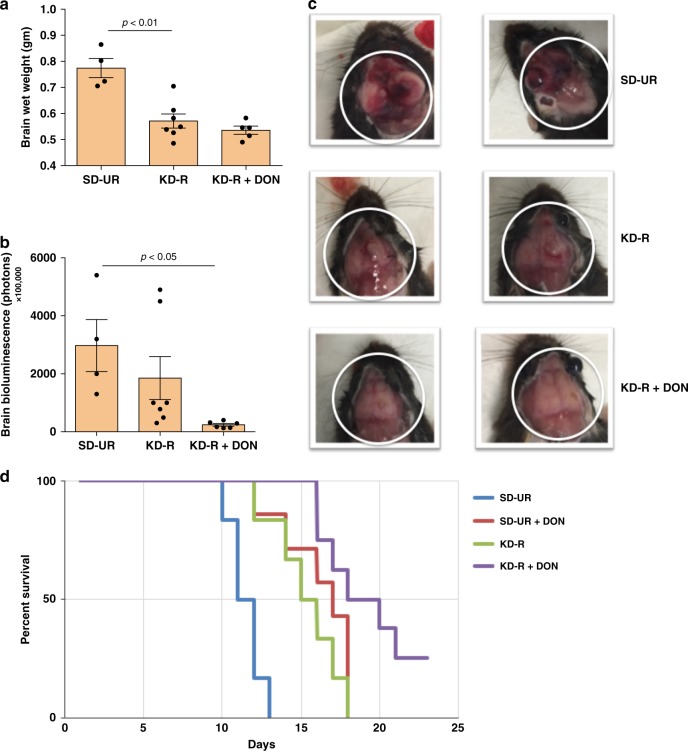
Restricted ketogenic diet with DON reduces progression and mortality of the CT-2A GBM. C57BL/6J mice were implanted with CT-2A tumour fragments on day 0, as described in Fig. 1. Brain wet weights were measured for SD-UR (*n* = 4 mouse brains), KD-R (*n* = 7 mouse brains), and KD-R + DON (*n* = 5 mouse brains) (**a**). Ex vivo bioluminescent photon values were calculated for the same brain samples and presented as the mean ± SEM (**b**). All brains were imaged on day 14 or 15. A one-way analysis of variance followed by Tukey's post hoc test was performed to determine the signicance between groups. Representative brain sample images from each study group were portrayed in (**c**). A Kaplan–Meier survival curve was plotted for the SD-UR (*n* = 6 mice), SD-UR + DON (*n* = 8 mice), KD-R (*n* = 6 mice), and KD-R + DON (*n* = 8 mice) groups (**d**). The log-rank test was used to determine the significance. Source data are provided as Supplementary Data 1

The CT-2A tumour is characterized as a highly angiogenic malignant mouse astrocytoma with stem cell characteristics^32,38^. The wet weight of the brain plus CT-2A tumour was lower in mice fed the KD-R than in mice fed the SD-UR, indicating a reduction of edema (Fig. 7a). Ex vivo bioluminescence of brains containing the CT-2A tumour was lower in mice fed the KD-R + DON than in mice fed the SD-UR indicating a reduction in the number of living CT-2A tumour cells in the brain (Fig. 7b). Representative mice with the CT-2A brain tumour are shown in Fig. 7c. The brains of mice bearing the CT-2A tumour that were fed the SD-UR were highly hemorrhagic, thrombotic, and edematous. In contrast, the brains of CT-2A bearing mice that were treated with KD-R + DON appeared normal without hemorrhage, thrombosis, or edema (Fig. 7c). All untreated SD-UR control mice containing the CT-2A tumour died between day 10 and 13 (Fig. 7d). In contrast, CT-2A-bearing mice treated with the diet/drug combination survived until day 24 and onwards. Histological analysis also showed that CT-2A tumour cell density, mitotic figures, and hemorrhage was less prevalent in the mice fed the KD-R than in the mice fed the SD-UR (Supplementary Fig. 3). The appearance of mitotic arrest or catastrophe was also seen in the CT-2A tumour of mice treated with the KD-R + DON (Supplementary Fig. 3).

## Discussion

This study documents the therapeutic action of glucose and glutamine targeting in the VM-M3 and CT-2A syngeneic mouse models of GBM. It is known that calorie restriction, ketogenic diets, and DON can reduce metabolites in the glycolytic and glutaminolysis pathways, respectively^33,39–41^. However, we showed that the KD-R + DON could rescue mice with these GBM tumours from late-stage orthotopic growth. The diet/drug therapy reduced brain swelling, hemorrhage, and morbidity. The KD-R alone reduced the VM-M3/Fluc cell invasion and proliferation as seen by both in vivo and ex vivo bioluminescent imaging of the brains, with Ki-67 staining, and with histology of the brain tissues. Moreover, the KD-R produced therapeutic ketosis (low GKI value) and reduced TNF-α and Iba-1 levels, suggesting reduced inflammation as compared to the SD-UR control brains. The ability of DON treatment to arrest tumour growth and promote survival late in disease progression suggests a potentially direct role for glutamine metabolism in VM-M3/Fluc growth and invasion. This finding is consistent with the importance of glutamine in the metabolism of mesenchymal cells from which the VM-M3 tumour is derived^4,37,42^. Although DON treatment alone was effective in reducing tumour growth and invasion in the control SD-UR mice, a synergistic effect was observed when DON was administered together with the KD-R, providing evidence for a diet/drug interaction. The findings also support the press-pulse therapeutic strategy for the metabolic management of cancer with the KD-R serving as a press therapy and DON serving as a pulse therapy^6^.

Interestingly, the level of DON in the brain tumour tissue was higher under KD-R feeding than under SD-UR feeding suggesting that the KD-R facilitates DON delivery to the brain. This is significant because it allowed for a lower therapeutic dose of DON when administered under a KD-R regimen. A similar observation was made previously in showing that a KD-R facilitated the delivery of the iminosugar, N-butyldeoxynojirimycin, across the blood–brain barrier in mice with Sandhoff disease^43^. Although the mechanism remains to be determined, these findings suggest that the KD has potential as a facilitator of drug delivery to the brain for a broad range of neuro-pathological conditions. It is also important to mention that our previous findings clearly demonstrated that caloric restriction can maintain the blood vessel integrity in the mouse CT-2A and the human U-87 GBM and thus reduce the leakiness of the neovasculature^44^. Interestingly, a recent observation by Gordon et al. showed that DON treatment promotes the recovery of blood–brain barrier integrity in the mouse model of cerebral malaria^33^. Further studies are needed to clarify the mechanism by which a KD-R facilitates drug delivery to the brain.

DON has shown therapeutic efficacy in human cancers of the blood, colon, and lung, but issues of toxicity were noted in some cases^30^. No noticeable toxicity was seen in the DON-treated mice until the end of the study when mild muscle loss was observed in some of the mice. It is proposed that issues with DON toxicity arise when there is an appreciable competition for glutamine between host and tumour. There is also increasing evidence supporting the protective role of glutamine supplementation for cancer patients^45^. Since tumour consumption of glutamine is dissipative, glutamine supplementation might not increase tumour growth^46^. However, additional preclinical studies will be needed to determine if glutamine supplementation could be used together with DON. It is our view that strategies for managing toxicity of a therapeutically effective drug would be as important as developing a new drug. Our results indicate that the KD-R enhances the therapeutic action of DON, thus reducing dosage and toxicity.

The therapeutic action of the diet/drug effect seen in the VM-M3 tumour was also seen in the CT-2A neural stem cell tumour. As observed from histological analysis, the KD-R + DON treatment caused massive mitotic arrest or catastrophe in the CT-2A brain tumour cells. The results show that therapeutic efficacy against orthotopic brain tumour growth was greater using the diet/drug combination than in using either the KD-R or DON alone. The CT-2A tumour shares several characteristics with glioma stem cells^32^, whereas the VM-M3 tumour shares several characteristics with the invasive mesenchymal cell found in most GBM^2^. Hence, our findings suggest that the KD-R + DON therapeutic strategy could potentially target the two most common neoplastic cell types found in human GBM^3^. Our recent human GBM case report provides support for our prediction^15^.

Several mechanisms could underlie the therapeutic action of the diet/drug therapy used to treat the VM-M3 and CT-2A tumours. The KD-R will simultaneously target the glycolytic and pentose phosphate pathways, which are upregulated in GBM^47–49^. We previously showed that calorie restriction and restricted ketogenic diets target the IGF-1, PI3K, AKT, and Hif-1α signaling pathways in the CT-2A tumour^38,50^. The down-regulation of these pathways would reduce angiogenesis, inflammation, and mTOR signaling, while enhancing tumour cell apoptosis^23,44,51,52^. As glucose is the fuel for glycolysis, the pentose phosphate pathway, and serine biosynthesis, the KD-R should reduce multiple growth metabolites, tumour cell glutathione levels, and nucleotide synthesis. Furthermore, the KD-R should also reduce one-carbon metabolism especially in glioma cells lacking OxPhos capacity^25,49,53^. Hence, the glucose-restricting action of the KD-R will target multiple signaling pathways linked to glioma growth and progression.

In addition to restricting glucose availability, the KD-R will also elevate β-hydroxybutyrate and acetoacetate, the major circulating ketone bodies produced in the liver, from the active metabolism of medium chain triglycerides^54^. Ketone bodies increase the redox span of the CoQ couple, thus reducing oxidative stress in normal brain cells^54^. These ketone bodies are also neuroprotective and have beneficial therapeutic value for various diseases^24,55^. As ketone bodies are non-fermentable and require efficient OxPhos for generating ATP^25,54^, the VM-M3 and CT-2A cells will be unable to metabolize these metabolites for energy due to insufficient OxPhos^12,25^. Ketone bodies cannot be used as an alternative energy fuel in cells with defective mitochondria^12,25^. Previous studies demonstrated that glioma cells cannot use ketone bodies and that ketone bodies do not stimulate tumour growth^56,57^. Hence, the KD-R has a dual function in (1) targeting glucose-dependent signaling pathways that drive tumour growth, and (2) in providing an alternative metabolic fuel to normal brain cells under glucose restriction.

While the KD-R will reduce metabolites through glycolysis and the pentose phosphate pathway, DON will block glutaminolysis, thus depriving the tumour cells of the amide nitrogen needed for nucleotide and protein synthesis, and at the same time, will deplete the glutamate needed for synthesis of α-ketoglutarate (α-KG)^25,58,59^. α-KG is a precursor for lipid synthesis through reductive carboxylation and is the substrate for ATP synthesis through the succinate-CoA ligase reaction in the TCA cycle under hypoxia^25,60^. As mentioned previously, however, glutamine targeting is more challenging than is glucose targeting due to the importance of glutamine for the immune system and the gut^6,61^. Consequently, glutamine targeting must be done strategically to avoid damage to those systems that are needed for normal physiological function^6,15^. It is for this reason that we administered DON to the tumour-bearing mice at low doses and transiently. We administered DON 7 days after tumour implantation, which is considered late for the VM-M3/Fluc tumour because neurological signs and symptoms start to appear 10–12 days post implantation followed by 100% death in 14–18 days. The late administration of DON was chosen to ensure adequately high ketone levels were achieved in order to facilitate neuroprotection and to reduce tumour inflammation. Secondly, the late treatment could serve as a comparable model when treating late-stage GBM patients. A 60–70% reduction in tumour growth was observed when DON was administered at 0.5 mg/kg once or twice per week. In comparison, a near complete resolution of the brain tumour was achieved when 1.0 mg/kg of DON was administered with the same schedule, as seen with bioluminescent imaging. These results are significant because no other clinical or pre-clinical study using DON considered a KD-R as a method for protecting the host from toxicity. Case reports of GBM and other metastatic cancers show the significance of the KD-R when combined with other therapies^15,62,63^.

Our findings support previous suggestions that reduced glutaminolysis would be a key therapeutic action of DON against the growth of glutamine-dependent tumours, including GBM^29,30^. Although it is known that DON targets glutaminolysis in tissues^30,33,58^ and that reduced levels of circulating glucose will reduce glycolysis^64,65^, we were unable to find consistent brain metabolite changes in pathways of glycolysis and glutaminolysis in a preliminary metabolomic analysis of KD-R + DON-treated VM-M3 mouse brains. Due to the confounding variables in the in vivo environment (dead tumour cells, necrotic tissue, and various types of host infiltrating cells), we believe that an in vitro analysis in defined media could provide a clearer picture of the changes in metabolites of the glucose and glutamine pathways due to DON or other drug treatments in the VM-M3 and CT-2A cells. It will also be important to determine if other glutaminolysis inhibitors, e.g., epigallocatechin-3-gallate (EGCG), bis-2-(5-phenylacetamido-1,2,4-thiadiazol-2-yl) ethyl sulfide (BPTEs), and CB-839^19,66^, can match the therapeutic action of DON against brain cancer when used together with the KD-R. Pretreatment with a ketogenic diet before implementing the standard of care is suggested in order to protect normal brain cells from treatment toxicities^22^.

It is important to mention that our findings in the VM-M3 and CT-2A syngeneic GBM models might be considered contradictory to some previous studies in GBM patients and in experimental systems^67–69^. Previous studies from Bachoo and colleagues suggest minimal ^13^C-glutamine anaplerosis in GBM implying that glutamine restriction in this context may not be therapeutic^68^. As DON inhibits multiple glutaminases, it is possible that DON does not directly impact glutamine anaplerosis in vivo, but acts on other related pathways and systems^29,30,33^. We recently described how glutamine is the only amino acid not requiring an energy investment to generate ATP through mitochondria substrate level phosphorylation (mSLP)^25^. Tardito et al. found that under glutamine starvation, intracellular oleate was unaffected, and glucose-dependent glutamate production increased, implying that the contribution of glutamine to growth is largely independent of anaplerosis^69^. Further, Oizel et al. identified two clusters of GBM that are glutamine^high^ and glutamine^low^ based on glutamine utilization^16^. These investigators also mentioned that the most aggressive GBM contained mesenchymal cells that utilized the highest levels of glutamine. The VM-M3 cells are highly invasive and of mesenchymal origin^2,37^. Nevertheless, we use caution in the interpretation of our data, as the metabolic effects of DON’s action in killing the GBM cells were inferred rather than measured directly.

**Fig. 8:**
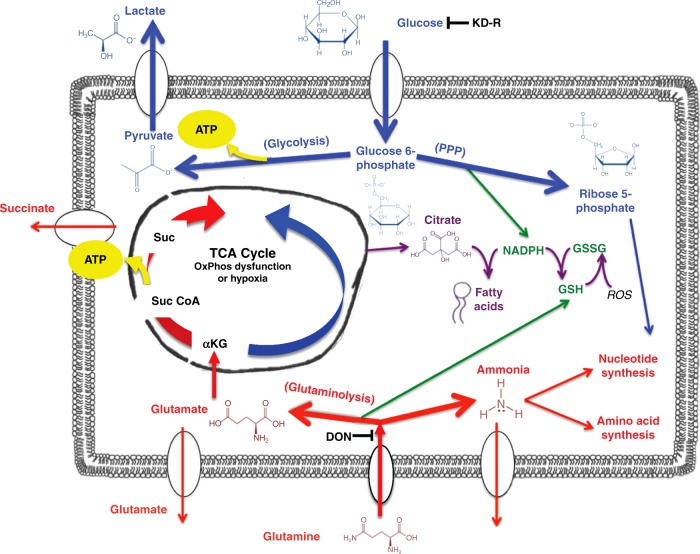
Targeting glucose and glutamine using KD-R with DON for the Metabolic Management of the VM-M3 and CT-2A Experimental GBM. GBM tumour cells are largely dependent on glucose and glutamine for survival and growth. Energy through substrate level phosphorylation (SLP) in the cytoplasm (glycolysis) and in the TCA cycle (glutaminolysis) will compensate for reduced energy through oxidative phosphorylation (OxPhos) or hypoxia that occurs in these GBM cells. The KD-R will reduce glucose carbons for both the glycolytic and pentose phosphate (PPP) pathways that supply ATP and precursors for lipid and nucleotide synthesis, as well as for glutathione production. DON will inhibit glutaminases thus depleting glutamate and the glutamine-derived amide nitrogen for ammonia and nucleotide synthesis. Depletion of glutamine-derived glutamate will reduce anapleurotic carbons to the TCA cycle through α-KG for protein synthesis, while also reducing ATP synthesis at the succinyl CoA synthase step in the TCA cycle. The glutamine-derived glutamate is also used for glutathione production that protects tumor cells from oxidative stress. The KD-R + DON will thus make the VM-M3 and the CT-2A cells vulnerable to oxidative stress. The simultaneous targeting of glucose and glutamine using the KD-R + DON will starve tumour cells of energy production while blocking their ability to synthesize proteins, lipids, and nucleotides. This metabolic starvation could also reduce extracellular acidification through reduction of lactate and succinate. The elevation of non-fermentable ketone bodies will provide normal cells with an alternative energy source to glucose while also protecting them from oxidative stress. This diagram and legend have been modified from that presented previously under the terms of the Creative Commons Attribution 4.0 International License in Nutrition and Metabolism by Thomas Seyfried et al. (http://creativecommons.org/licenses/by/4.0/)^6^

In the present study, we demonstrate the benefits of targeting glutamine under a KD-R for managing experimental GBM. The influence of targeting both glucose and glutamine on the metabolic pathways involved with GBM growth is shown in Fig. 8. This treatment was capable of arresting the growth of tumour cells and in promoting the survival of mice with two different syngeneic GBM tumours grown orthotopically. As the mice used for these studies were not treated with surgery, radiation, or standard chemotherapy, it is unclear if a similar therapeutic response would be seen in GBM patients using this approach together with current standard of care^15^. Because of the high mortality following a GBM diagnosis, our findings should have clinical implications especially in light of our recent case report^15^. Furthermore, our studies reveal a potential role for the ketogenic diet in facilitating drug delivery to brain tumours.

## Materials and methods

### Mice

Mice of the VM/Dk (VM) strain were obtained as a gift from H. Fraser (University of Edinburgh, Scotland). The C57BL/6J (B6) mice were obtained originally from the Jackson Laboratory, Bar Harbor, ME. All mice used in this study were housed and bred in the Boston College Animal Care Facility using IACUC approved husbandry conditions^28^. Male and female mice between 8–10 weeks of age were used for all studies. All animal procedures and protocols were in strict accordance with the NIH Guide for the Care and Use of Laboratory Animals and were approved by the Institutional Animal Care Committee at Boston College under assurance number A3905–01.

### The VM-M3 and CT-2A murine GBM models

The VM-M3 tumour used in this study arose spontaneously in the cerebrum of an adult male mouse of the VM/Dk inbred strain. A cell line was prepared from the VM-M3 tumour^37^. The VM-M3 tumour manifests all of the invasive characteristics seen in human GBM^2^. The CT-2A tumour was originally produced from implantation of 20-methylcholanthrene into the cerebral cortex of a C57BL/6J mouse and was broadly classified as a poorly differentiated highly malignant anaplastic astrocytoma and a cell line was produced from this tumour^31^. Both cell lines are mycoplasma free and we use MycoZap (Lonza) kit to eradicate mycoplasma. More recent studies have classified the CT-2A tumour as a neural stem cell tumour^32^. The VM-M3 and the CT-2A cell lines were transduced with a lentivirus vector (gift from Miguel Sena-Esteves) containing the firefly luciferase gene under control of the cytomegalovirus promoter (VM-M3/Fluc) to produce VM-M3/Fluc and CT-2A/Fluc cell lines^37^. This transduction allows the cells to be tracked in the brain using bioluminescent imaging.

### Tumour implantation

Tumour implantation was performed using our standard protocol^2^. Briefly mice were anaesthetized with isoflurane (5% in oxygen). The tops of the heads were disinfected with ethanol and a small incision was made in the scalp over the midline. A 3 mm^3^ burr hole was made in the skull over the right parietal region behind the coronal suture and lateral to the sagittal suture. Small (1 mm^3^) tumour fragments were implanted approximately 1.5–2.0 mm deep into the cortical region using a trocar, as previously described. The skin flaps were closed with 7 mm reflex clips. The mice were placed in a warm room (24 °C) until they were fully recovered. The procedure confirms 100% recovery within a few hours of implantation. The GBM tumour cells are highly invasive regardless of implantation method, and all tumour-implanted mice reach morbidity at approximately 12–15 days.

### Dietary/drug regimens and body weight

All mice received the standard diet unrestricted (SD-UR) prior to initiation of the study. Tumour fragments were implanted on day zero. Upon implantation of the tumour, mice remained on the SD-UR until a 15-h fast was initiated 72 h after implantation. Following the fast, mice were introduced to the Ketogenic diet restricted (KD-R), or remained on the SD-UR, depending on the study group. The KD-R has a caloric density of 7.12 Kcal/g. The percent nutritional breakdown of KetoGEN (Medica Nutrition, Canada) is as follows: 2.1% of Cal from carbohydrates, 8.7% of Cal from protein, and 89.2% of Cal from fat (Supplementary Table 1). The Medica Nutrition cancer ketogenic diet that we used was prepared without added vitamins and minerals. Previous studies by Tannanbaum showed that the therapeutic effects of calorie-restricted diets come largely from the restriction of macronutrients and not from restriction of micronutrients^70^. Mice were individually housed beginning at the initiation of the 15 h fast. Food intake for the KD-R mice was measured to be between 1–3 g per day to maintain a 15–18% body weight reduction in each mouse. Mice were weighed daily to ensure weight maintenance (Supplementary Fig. 4).

Mice receiving the SD-UR were given the standard chow diet (Lab Diet) ad libitum for the duration of the study. For those mice that received DON injections, a fresh stock was prepared and diluted to an appropriate concentration in PBS and was administered intraperitoneally (i.p.). The DON stock solution in PBS was stored at −20 °C for the duration of the study and mice received 200 μl injections of 0.1–1.0 mg/kg. Mice in the DON survival study received DON at a dosage that was appropriate for the individual mouse response. Some doses were skipped if the mice appeared lethargic or if body weight loss exceeded 1.5 g from the previous day. Studies were terminated at the time of morbidity for the control SD-UR group.

### Bioluminescence imaging

The Xenogen IVIS system is used to record the bioluminescent signal from the labeled tumours as we previously described^37^. Briefly, for in vivo imaging, mice received an i.p. injection of d-lucifierin (50 mg/kg) in PBS and Isofluorane (5% in oxygen). Imaging times ranged from 1 to 5 min, depending on the time point. For ex vivo imaging, brains were removed and imaged in 0.3 mg d-luciferin in PBS. The IVIS Lumina cooled CCD camera system was used for light acquisition. Data acquisition and analysis was performed with Living Image software (Caliper LS).

### Histology

Brain tumour samples were fixed in 10% neutral buffered formalin (Sigma) and embedded in paraffin. The brain tumour samples were sectioned at 5 μm, were stained with haematoxylin and eosin (H&E) at the Harvard University Rodent Histopathology Core Facility (Boston, MA), and were examined by light microscopy using either a Zeiss Axioplan 2 or Nikon SMZ1500 light microscope. Images were acquired using SPOT Imaging Solutions (Diagnostic Instruments, Inc.) cameras and software. All histological sections were evaluated at the Harvard University Rodent Histopathology Core Facility.

### Immunohistochemistry: Ki-67 and Iba-1

For immunohistochemistry, the tissue sections from untreated and treated tumour-bearing mice were deparaffinized, rehydrated, and washed. The tissue sections were then heat treated (95 °C) in antigen unmasking solution (Vector Laboratories, Burlingame, CA) for 30 min. Tissue sections were blocked in goat serum (1:10 in PBS) for one hour at room temperature, treated with Ki-67 primary antibody (rat monoclonal, Dako, 1:100) overnight at 4 °C followed by Alexafluor conjugated anti-rat secondary antibody at 1:100 dilution. Nuclei were stained using NucBlue (Invitrogen). Images were captured by the EVOS FL Cell Imaging System fluorescent microscope. For Iba-1, tissue sections were blocked with Iba-1 primary antibody (rabbit monoclonal, abcam, 1:1000) overnight at 4 °C followed by biotinylated anti-rabbit secondary at 1:2000 dilution. Avidin-biotin reaction was complete by incubating the tissues in ABC reagent (Vector Lab) for 30 min followed by DAB substrate reaction. Methyl green was used for staining the nucleus.

### Western blot analysis of Iba-1 protein expression

Frozen tumour and normal brain tissues were homogenized in ice-cold lysis buffer containing 20 mmol/L Tris-HCl (pH 7.5), 150 mmol/L NaCl,1 mmol/L Na_2_EDTA, 1 mmol/L EGTA, 1% Triton, 2.5 mmol/L NaPPi,1 mmol/L a-glycerophosphate, 1 mmol/L Na3PO4, 1 Ag/mL leupeptin, and 1 mmol/L phenylmethylsufonyl fluoride.

Lysates were transferred to 1.7 mL Eppendorf tubes, mixed on a rocker for 1 h at 4 °C, and then centrifuged at 8100*xg* for 20 min. Supernatants were collected and protein concentrations were estimated using the Bio-Rad detergent-compatible protein assay.

Approximately 100 μg of total protein from each tissue sample was denatured with SDS-PAGE sample buffer [63 mmol/L Tris-HCl (pH 6.8), 10% glycerol, 2% SDS, 0.0025% bromphenol blue, and 5% 2-mercaptoethanol] and was resolved by SDS-PAGE on 4 to 12% Bis-Tris gels (Invitrogen). Proteins were transferred to a polyvinylidene difluoride immobilon TM-P membrane (Millipore) overnight at 4 °C and blocked in either 5% nonfat powdered milk or 5% bovine serum albumin in TBS with Tween 20 (pH 7.6) for 1–3 h at room temperature. Membranes were probed with primary antibodies (Iba-1, abcam, UK) overnight at 4 °C with gentle shaking. The blots were then incubated with the appropriate secondary antibody (anti-rabbit) for 1 h at room temperature and bands were visualized with enhanced chemiluminescence. Each membrane was stripped and reprobed for β-actin as an internal loading control and the ratio of the Iba-1 to β-actin was analyzed by scanning densitometry (FluorChem 8900 Software).

### Liquid chromatography mass spectrometry analysis of DON

Two independent LC-MS instruments and procedures were used to measure the amount of DON in the blood and in the VM-M3 tumour tissues. This was done to validate the accuracy of the procedures. A Triple quad method (Agilent 6460) was used to analyze DON in the first procedure (Fig. 4a). Mice were injected with 1.0 mg/kg of DON at either 10 or 60 min before collection of the brains and blood. The brains were immediately flash frozen. Blood samples were collected before removal of the brain. 3 N HCl + n-butanol (250 μl) was added to 50 μl of plasma, vortexed and then centrifuged at 16,000*xg* for 5 min to precipitate proteins. A 200 μl aliquot of the supernatant was transferred to a 1.5 ml tube and incubated at 60 °C for 30 min in a shaking water bath to perform the DON derivatization reaction. For the brain tissue, 5 μl of n-butanol containing 3 N HCl was added per milligram tissue. The tissue sample was then homogenized with a glass pestle and was then vortexed and centrifuged, and prepared similarly to the plasma samples. Standard solutions were prepared by serial dilution to generate concentrations from 10 to 100 μM. DON was added to untreated mouse plasma or brain tissue to generate an internal standard curve. A high resolution LC-MS QToF method (Agilent 6550) was used to analyze DON in the second procedure (Fig. 4b). Mice were injected with DON as above. Pre-weighed frozen brain tissue was transferred to a tube containing ceramic beads (Omni tubes). 3M HCl + butanol was added at a concentration of 5 μl/mg of tissue. The sample was then homogenized via a bead ruptor homogenizer for 45 s, let rest for 30 s, and homogenized again with the bead ruptor for an additional 45 s. Subsequently, the homogenate was derivatized by incubating at 60 °C for 30 min. The homogenate was then centrifuged at 16,000*xg* for 10 min at 4 °C. The supernatant was transferred to a fresh 1.5 ml tube and was centrifuged again to remove excess debris. A 200 μL aliquot of the supernatant was dried in a centrifugal evaporator. The samples were resuspended in 50 μl dH_2_O containing 0.2% formic acid. A 5 μl aliquot of this solution was injected for DON analysis using a Phenomenex Kinetix 2.6 µM F5 column for an 8 min gradient. Standard curves were also used as described above.

### Blood glucose and ketone measurements

All mice were fasted for 2 h before blood collection to stabilize blood glucose levels. Blood glucose and ketone levels were measured using the Keto-Mojo monitoring system (keto-mojo, Napa, California). Whole blood from the tail was placed onto the glucose or ketone strip. The keto-mojo meter was used to determine the mmol levels of glucose and β-hydroxybutyrate in the blood. The GKI was determined as we previously described^36^.

### Tumour necrosis factor determination

VM-M3 brain tumour tissue and normal brain tissue from the VM/Dk mice were homogenized and processed following the Quantikine Mouse TNF-α Immunoassay protocol (R&D systems). TNF-α levels were measured in triplicate and adjusted with the protein level of each sample. The limit of detection is <10 pg/ml Tumour necrosis factor (TNF).

### Statistics and reproducibility

Body weight, food intake, tumour growth, and plasma metabolite levels were analyzed using the one-way analysis of variance (ANOVA) followed by Tukey’s post hoc test or by a Student’s *t*-test to perform a two-sided pairwise comparison among the groups (SPSS 14.0). In each figure, error bars are mean ± SEM and *n* is the number of individual mice analyzed. The Survival studies were plotted on a Kaplan–Meir curve using Graph Pad Prizm software and significance was determined using the log-rank test.

### Reporting summary

Further information on research design is available in the Nature Research Reporting Summary linked to this article.

## Supplementary information

supplementary file

Reporting Summary

Description of Supplementary Data

Supplementary Data 1

## Data Availability

The authors declare that all data supporting the findings of this study are available upon request from the corresponding author. The source data for all figures are provided as Supplementary Data 1.
