# Supplementary material for: Therapeutic benefit of combining calorie-restricted ketogenic diet and glutamine targeting in late-stage experimental glioblastoma
Source: Commun Biol. 2019 May 29;2:200. doi: 10.1038/s42003-019-0455-x (PMC6541653; doi:10.1038/s42003-019-0455-x)
Supplement: Supplementary file 1 — supplementary file [file 42003_2019_455_MOESM1_ESM.pdf]

Purna Mukherjee et al

Supplementary Figure 1

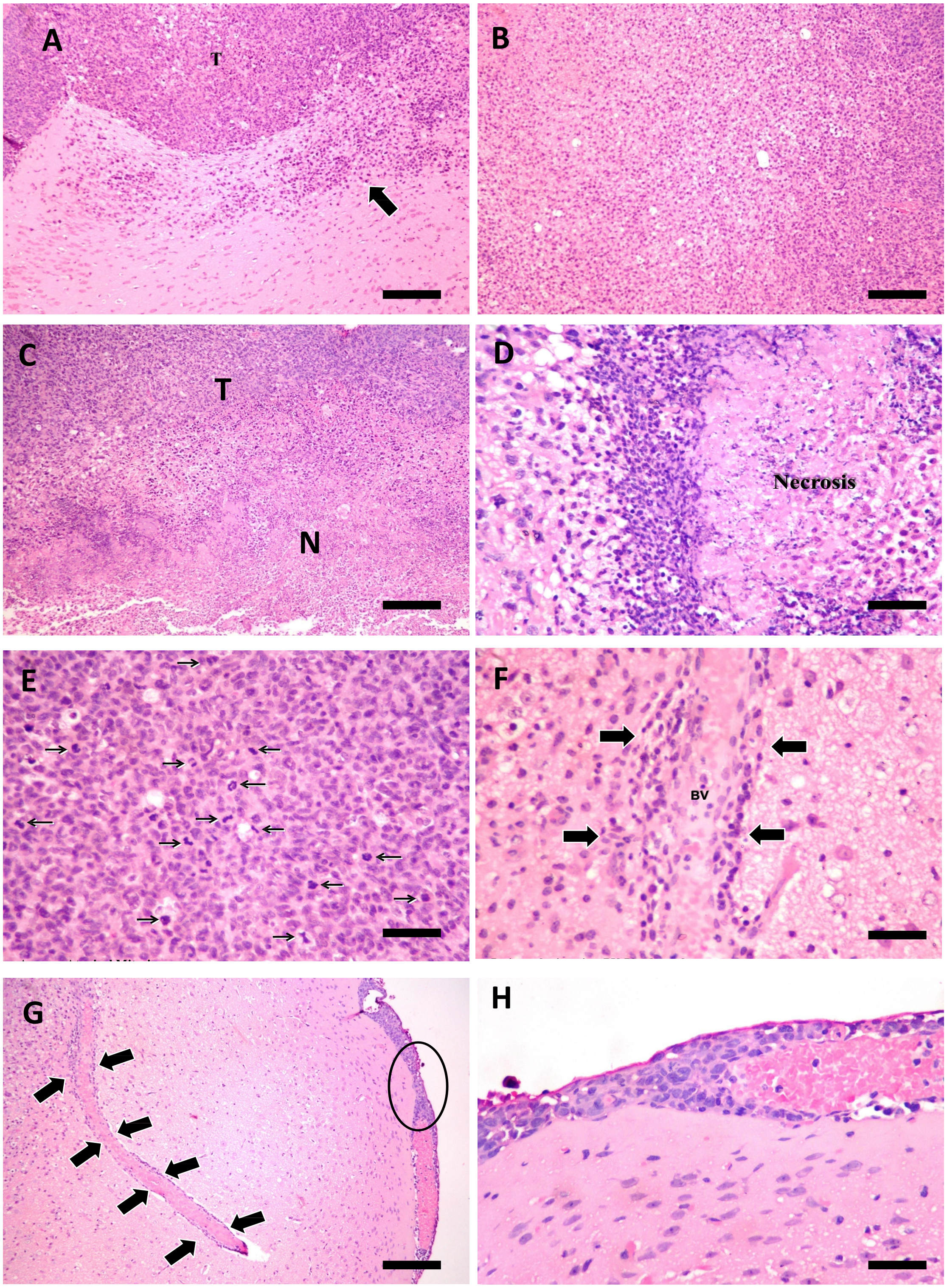

Invasive behavior in brain of VM-M3 GBM tumour cells. VM-M3/Fluc tumor fragments were implanted as described in **Figure 1**. Histological analysis (H&E) was used to validate the presence of tumour cells in the different regions of the brain as indicated in the figures. A defining characteristic of GBM is the secondary structures of Scherer, which is evident in panels A-H. **Panel A**: tumour cell infiltration in the brain (scale bar = 175 µm). **Panel B**: core tumor area (scale bar = 300 µm). **Panel C**: tumor (T) and necrotic (N) areas (scale bar = 525 µm). **Panel D**: necrosis in higher magnification (scale bar = 140 µm). **Panel E**: arrows indicate atypical mitosis (scale bar = 100 µm). **Panel F**: arrows show perivascular invasion (scale bar = 65 µm). **Panel G**: arrows show perivascular invasion and circle shows sub-arachnoid invasion (scale bar = 200 µm). **Panel H**: subarachnoid space infiltration in higher magnification (scale bar = 65 µm).

Supplementary Figure 2

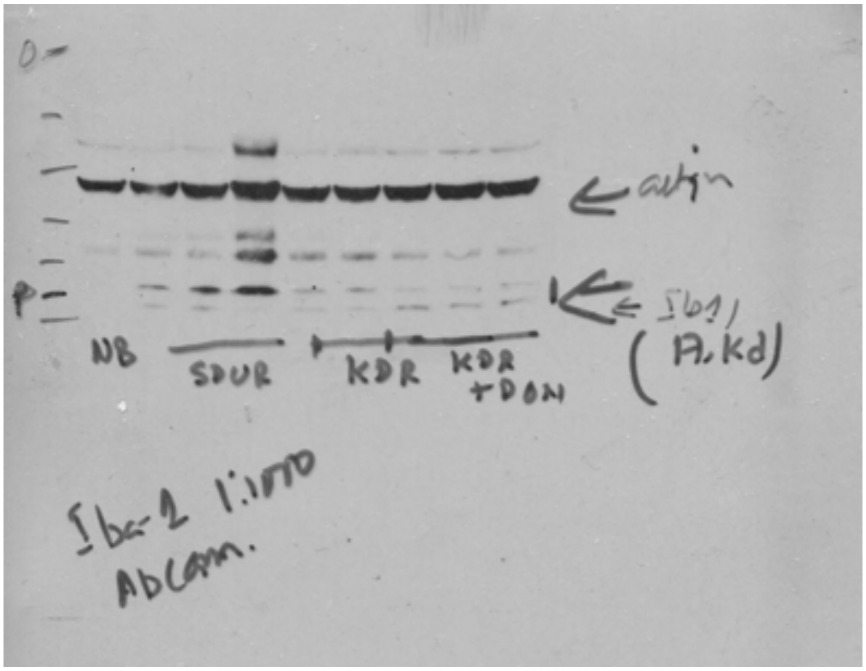

Original immunoblot for panel figure 6b showing KD-R with DON reduces Iba-1 in VM-M3 brain tumour tissue. The expression of Iba-1 (17kd) decreased in the DON treated tumours (n=3 mouse tumour brain tissue) in comparison to SD-UR (n=3 mouse tumour brain tissue). A decrease in expression was also seen for KD-R mice (n=2 mouse tumour brain tissue). Normal brain (NB) was used as a negative control tissue. Actin was used on the same membrane as a loading control. No image alterations were performed on original immunoblots other than cropping to create the figures in the manuscript.

Supplementary Figure 3

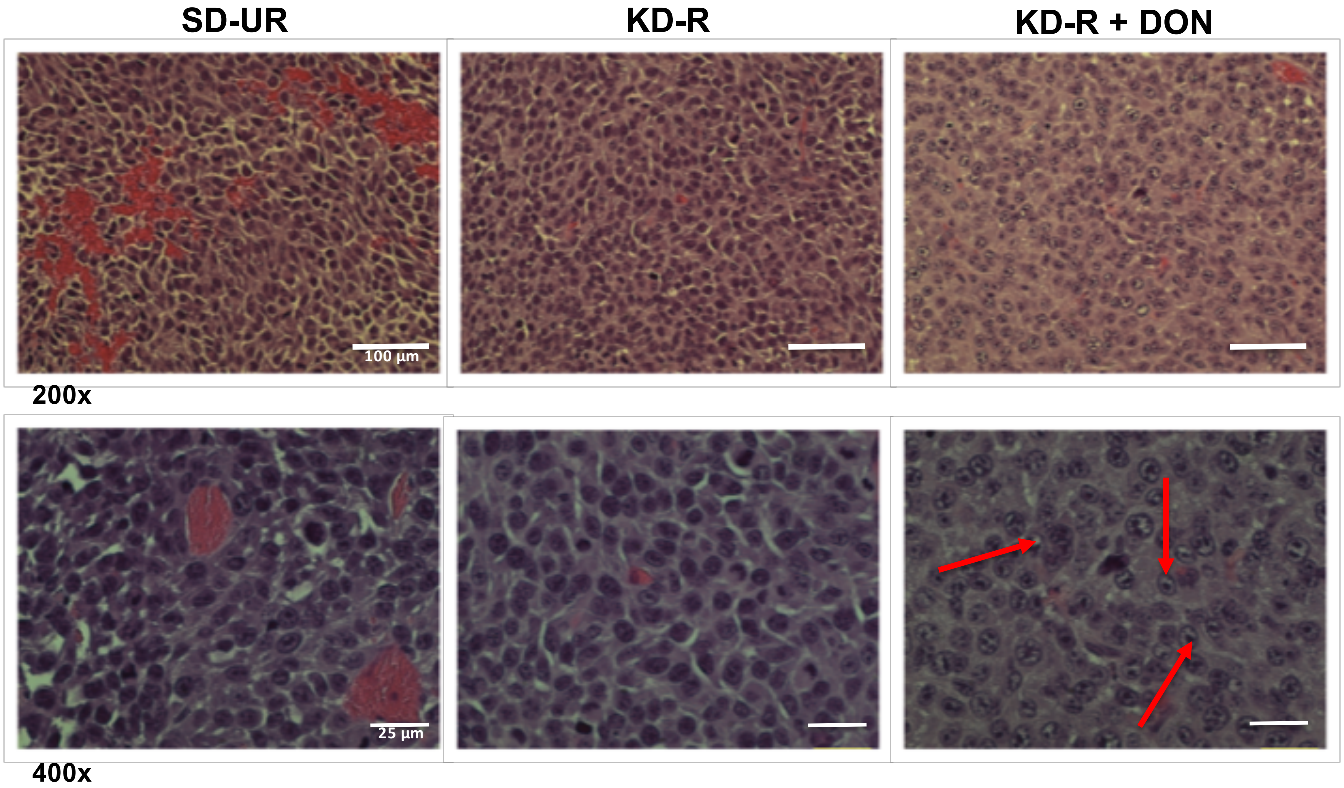

Histological analysis of KD-R and DON treated CT-2A tumour brains. H&E was used to validate the influence of the KD-R and DON treatment on the CT-2A tumor cells. Images show the tumor core at 200 x (top), and at 400 x (bottom). Arrows indicate nuclear mitotic arrest. All scale bars are 100 µm for 200x and 25µm for 400x.

Supplementary Figure 4

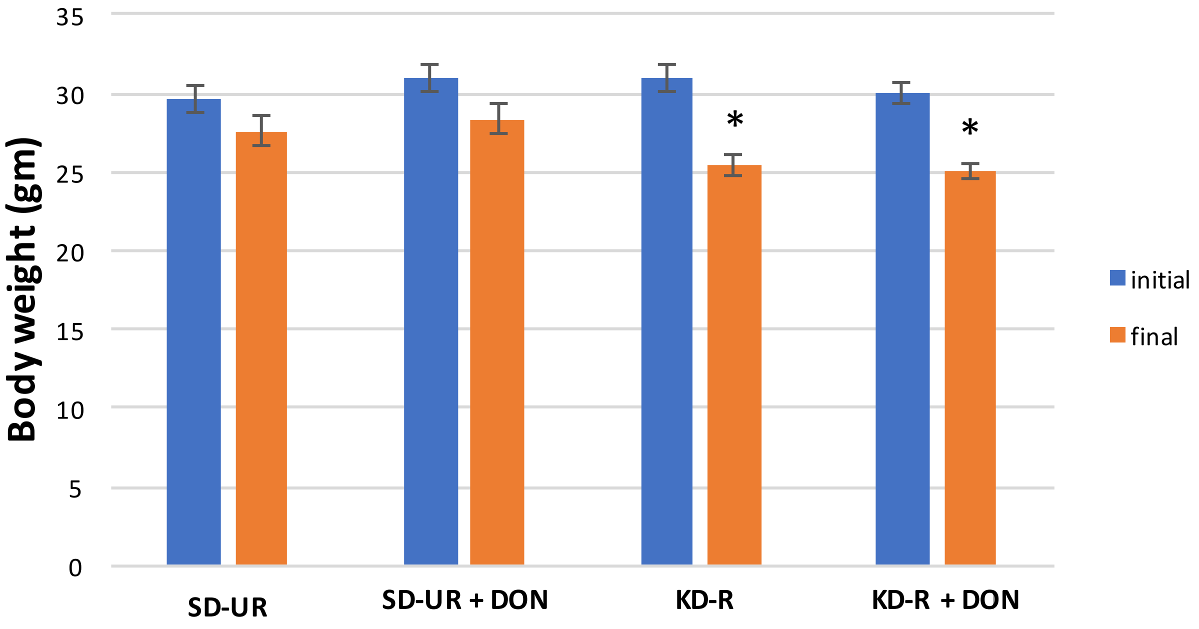

Body weights of VM-M3 tumour bearing mice. VM-M3/Fluc tumour fragments were implanted as described in Figure 1. Body weight was determined every alternate day. Initial and final body weight data are presented. Mice fed the SD-UR began to drop body weight on the day of termination due to tumour burden. Mice fed the KD-R maintained a 15-18 % body weight reduction throughout the experiment due to reduced caloric intake. Initial and final body weights were similar in the SD-UR and the SD-UR + DON mice (n = 15 mice/group). Final body weights were lower than initial body weights in the KD-R and the KD-R + DON mice (n=15 mice/each group). Values are expressed as the mean +/- SEM and a two tailed student's t-test was performed between the initial and final values of each group (* = p<.01).

Supplementary Table 1

Composition (%) of the standard diet and the ketogenic diet		
**Components**	**Standard Diet**	**Ketogenic Diet**
Carbohydrate	62	3
Fat	6	72
Protein	27	15
Energy (Kcal/g)	4.4	7.2
