# Supplementary material for: Therapeutic benefit of combining calorie-restricted ketogenic diet and glutamine targeting in late-stage experimental glioblastoma
Source: Commun Biol. 2019 May 29;2:200. doi: 10.1038/s42003-019-0455-x (PMC6541653; doi:10.1038/s42003-019-0455-x)
Supplement: Supplementary file 2 — Reporting Summary [file 42003_2019_455_MOESM2_ESM.pdf]

nature research

Corresponding author(s): Thomas Seyfried

Last updated by author(s): 2019/04/23

Reporting Summary

Nature Research wishes to improve the reproducibility of the work that we publish. This form provides structure for consistency and transparency in reporting. For further information on Nature Research policies, see Authors & Referees and the Editorial Policy Checklist.

Statistics

For all statistical analyses, confirm that the following items are present in the figure legend, table legend, main text, or Methods section.

n/a	Confirmed	
☐	☒	The exact sample size (*n*) for each experimental group/condition, given as a discrete number and unit of measurement
☐	☒	A statement on whether measurements were taken from distinct samples or whether the same sample was measured repeatedly
☐	☒	The statistical test(s) used AND whether they are one- or two-sided<br>*Only common tests should be described solely by name; describe more complex techniques in the Methods section.*
☒	☐	A description of all covariates tested
☐	☒	A description of any assumptions or corrections, such as tests of normality and adjustment for multiple comparisons
☐	☒	A full description of the statistical parameters including central tendency (e.g. means) or other basic estimates (e.g. regression coefficient) AND variation (e.g. standard deviation) or associated estimates of uncertainty (e.g. confidence intervals)
☐	☒	For null hypothesis testing, the test statistic (e.g. *F*, *t*, *r*) with confidence intervals, effect sizes, degrees of freedom and *P* value noted<br>*Give P values as exact values whenever suitable.*
☒	☐	For Bayesian analysis, information on the choice of priors and Markov chain Monte Carlo settings
☒	☐	For hierarchical and complex designs, identification of the appropriate level for tests and full reporting of outcomes
☒	☐	Estimates of effect sizes (e.g. Cohen's *d*, Pearson's *r*), indicating how they were calculated

Our web collection on statistics for biologists contains articles on many of the points above.

Software and code

Policy information about availability of computer code

Data collection	excel, Living Image software (Caliper LS).
Data analysis	excel, Graph Pad- prizm, image J, Axiovision, Living Image software (Caliper LS).

For manuscripts utilizing custom algorithms or software that are central to the research but not yet described in published literature, software must be made available to editors/reviewers. We strongly encourage code deposition in a community repository (e.g. GitHub). See the Nature Research guidelines for submitting code & software for further information.

Data

Policy information about availability of data

All manuscripts must include a data availability statement. This statement should provide the following information, where applicable:

- Accession codes, unique identifiers, or web links for publicly available datasets
- A list of figures that have associated raw data
- A description of any restrictions on data availability

Fig 1b , c,d,f ; Fig 2a and 2b; Fig 4a and b; Fig 5a and b; Fig 6b; Fig 7a, b and d; s Fig 2 contain raw data   The authors declare that all data supporting the findings of this study are available within the paper [and its supplementary information files], available upon request from the corresponding authors. The source data for all figures are provided as a
Source Data file.

Field-specific reporting

Please select the one below that is the best fit for your research. If you are not sure, read the appropriate sections before making your selection.

☒ Life sciences ☐ Behavioural & social sciences ☐ Ecological, evolutionary & environmental sciences

For a reference copy of the document with all sections, see nature.com/documents/nr-reporting-summary-flat.pdf

Life sciences study design

All studies must disclose on these points even when the disclosure is negative.

Sample size	sample sizes were chosen based on previous similar studies that had statistically significant outcomes.
Data exclusions	No data was excluded from the study
Replication	all findings were replicated multiple times. All attempts at replication were successful
Randomization	Mice were randomly assigned to groups
Blinding	histology data was analyzed blindly

Reporting for specific materials, systems and methods

We require information from authors about some types of materials, experimental systems and methods used in many studies. Here, indicate whether each material, system or method listed is relevant to your study. If you are not sure if a list item applies to your research, read the appropriate section before selecting a response.

Materials & experimental systems

n/a	Involved in the study	
☐	☒	Antibodies
☐	☒	Eukaryotic cell lines
☒	☐	Palaeontology
☐	☒	Animals and other organisms
☒	☐	Human research participants
☒	☐	Clinical data

Methods

n/a	Involved in the study	
☒	☐	ChIP-seq
☒	☐	Flow cytometry
☒	☐	MRI-based neuroimaging

Antibodies

Antibodies used	Ki-67 - rat monoclonal cross react with mouse , DAKO (cat# M7249; clone Tec-3) , 1:500 for immunofluorescence for the tissue. Alexafluor conjugated secondary from in vitrogen. Iba-1 , rabbit monoclonal from abcam (cat#ab177846; lot# GR3235737-4), 1:500 for IHC of the tissue. Anti rabbit biotin conjugated secondary antibody. for the western blot, HRP
Validation	Both antibodies are widely used and validated. We used ki67 previously for immunohistochemistry and published. Iba-1 is validated for both immunohistochemistry and western blot for mouse tissue.

Eukaryotic cell lines

Policy information about cell lines

Cell line source(s)	VM-M3 cell line - mouse spontaneous GBM cell line grown in VM mice and CT-2A- mouse astrocyte cell line grown in C57BL/6 mice
Authentication	both cell lines are originated in this lab , cell line established and and authenticated. Other researchers are using these cell lines too.
Mycoplasma contamination	. Both cell lines were tested for mycoplasma contamination and there are no mycoplasma contamination in M3 and CT-2A cell lines
Commonly misidentified lines (See ICLAC register)	*no misidentified cell lines were used.*

Animals and other organisms

Policy information about studies involving animals; ARRIVE guidelines recommended for reporting animal research

Laboratory animals	VM male and female mice (8-10 weeks of age) were used for the study, tumor passage and breeding in the BC animal facility. C57BL/6 malefemale mice (8-10 weeks of age) were used for the study , tumor passage and breeding.
Wild animals	study did not involve wild animals.
Field-collected samples	study did not involve samples collected from the field
Ethics oversight	All animal procedures were in strict accordance with the NIH Guide for the Care and Use of Laboratory Animals and were approved by the Institutional Animal Care Committee at Boston College.

Note that full information on the approval of the study protocol must also be provided in the manuscript.
