# Supplementary material for: Therapeutic benefit of combining calorie-restricted ketogenic diet and glutamine targeting in late-stage experimental glioblastoma
Source: Commun Biol. 2019 May 29;2:200. doi: 10.1038/s42003-019-0455-x (PMC6541653; doi:10.1038/s42003-019-0455-x)
Supplement: Supplementary file 3 — Description of Supplementary Data [file 42003_2019_455_MOESM3_ESM.docx]

**Description of Additional Supplementary Files**

**File Name**: Supplementary Data 1

**Description**: Source data file
